# Surgery of the Antrum

**Published:** 1892-04

**Authors:** G. S. Junkerman


### Surgery of the Antrum.

BY G. S. JUNKERMAN, M.D., D.D.S.

This paper has nothing new to offer on the subject of the Antrum. It trusts rather to its kind reception before this Society on a weakness of human nature, viz.: that we always like to hear something that we know already; we like to discover that we know something that somebody else considers worthy of study.

The Antrum is only one of the air cells of the skull, and since it is of importance as the largest of these, it is known by the dignified title of the Antrum of Highmore. It like the other air cells of the skull, is lined by the Schneiderian membrane. It is of significance to the oral surgeon because it comes within his jurisdiction to treat its diseases, and it is the only air cavity that bears any relationship with the teeth. The Antrum is located mainly within the superior maxillary bone, but its walls are completed only by the addition of the inferior turbinated, lachrymal and palate bones, which bones are interested in forming most of its nasal wall. It is triangular of shape with its apex pointing to the canine fossa. The base looks backward toward the palate. The other walls look respectively toward the nasal fossa, the orbit and the palatine process of the superior maxillary bone. If this cavity has any function I think it differs somewhat from the function of the remaining air cells of the head, viz.: to warm the air before it passes to the lungs. In order to maintain the contour of the face nature has combined in this bone by the formation of this air cavity both lightness of weight and extent of surface. She has furnished an ample frame for the face and yet retained the balance of weight in the poise of the head. If the cavity were solid bone tissue it would make a remarkable difference in the weight of the head. There is another feature of importance which is the better maintenance of blood supply to the face, and of direct importance to the dental surgeon in that by the presence of this cavity a freer supply of blood and nerve force to the molars and pre-molars is maintained. All diseases of this cavity would be treated surgically. Yet we would not wish to magnify the importance of the diseases connected with it. It is subject to about the same diseases that may affect any other mucous membrane. Add to this the fact of its being a cavity of some obscurity and rather restricted walls, and we have in view all the obstacles with which we have to contend in diagnosis and treatment. As a matter of fact it may be stated that any Antral disease attains importance not as regards the Antrum itself, but as the progress of the disease affects the surrounding tissues. In most cases while the disease is confined to its own walls there is little or no inconvenience to the patient; but should it press upward the orbital cavity is invaded. If the disease should extend at the expense of the inner wall of the cavity the nasal fossa would be invaded, and should the floor of the cavity give way the mouth would be associated with the disease. A forward projection of the disease would involve the face. From this we find that the oral orbital and the nasal cavities are not only liable to be connected in disease with the Antrum, but owing to the thinness of the partition walls between these cavities they are very easily invaded.

For purpose of diagnosis and treatment of any disease of the Antrum, access to the cavity must be gained. There are three ways of entering the Antrum. There are two ways practical to the oral surgeon and both of these are by way of the mouth. These are by way of the canine fossa, which is a little depression just posterior to the root of the superior canine; and by way of the Alveolar cavities of the pre-molars and molars. The third entrance is by way of the middle meatus of the nasal fossa. This in reality, is the only entrance which in the recent state is open. The dental surgeon who is not equipped with instruments for treatment through this opening had better restrict himself to the other means of egress. Entrance should be gained into the Antrum by way of the canine fossa when the apex of the cavity can be definitely located. The disease may indicate the position of this opening, and under the circumstances you are perfectly justified in performing with a steel instrument and enlarging the opening to a convenient size. This is especially indicated if the teeth are all present and found to be intact. Engorgement of the Antrum characterized by facial tumor is the indication for this plan of procedure. Where facial tumor is not present there is no absolute rule by which you can reach the Antrum through the canine fossa. The development of the Antrum is so variable that it can not be perfectly determined just how far forward the cavity is developed. It may be as far forward as the canine or no farther than the first molar. There is little risk in forcing entrance through the palatine root of the second molar; and this means should always be resorted to if possible. Facial tumor and preservation of the teeth would indicate the canine fossa operation. The aveolar operations are preferable in all cases where the preservation of the teeth is not concerned. It would be of no purpose to enumerate the various diseases which may afflict the Antrum and indicate their treatment. These points of information may be gained by consulting any chapter on mucous membranes and their diseases. With the Antrum the diseases are complicated by their being confined to a cavity. Free and open drainage for the cavity is the first principle of treatment; antisepsis the other principle of treatment.

The operator is frequently brought in contact with the Antrum accidentally in the process of the extraction of teeth.

This may occur by the forcing of the root into the Antral cavity, thereby fracturing the alveolar wall; or by tearing away part of the floor of the Antrum with the root of the tooth. Either case is liable to produce a complication in the form of troublesome inflammation. The former mishap would necessitate the enlarging of the opening made, and a removal, if possible, of the foreign body. If this could not be accomplished the opening should be enlarged to give the products of inflammation a chance to expel it. There are, no doubt, many obscure pains, especially of a neuralgic nature about the head and face that arise from Antral trouble, and a small amount of surgical interference will often relieve a seeming severe complication.

There are diseases of the Antrum that are brought by association with the other air cavities, amenable to treatment through the nasal opening of the Antrum, but these are not in the field of the oral surgeon.

---

DISCUSSION ON DR. G. S. JUNKERMAN'S PAPER.

*Dr. Wm. Knight.*—The essayist has read an interesting paper on a very interesting subject. As far as its anatomy is concerned, in addition to the antrum being like the ordinary air cells of the head, its purpose seems to me to add largely to the resisting power of the upper maxillary bone. It is a well-known fact in mechanics that, the same quantity of material being used, a hollow cylinder is much stronger than a solid one, and this seems to be one purpose for the superior maxillary bone being hollow, to meet the resistance of the lower jaw.

The mucous membrane lining the antrum is in some sense more subject to disease than the neighboring nasal mucous membrane; it is exposed to the ordinary influences of disease, likewise subject to injury from decayed roots of teeth opening into the cavity.

As regards the affections to which this cavity is liable, Empyema is the most frequent, and this affection does not produce expansion of the walls, except in serious and prolonged cases; it is more readily recognized by deep-seated pain referred to the upper part of the cheek, and by occasional discharges from the corresponding side of the nose, and from a peculiar odor noticeable only to the patient, but not to his friends. A cystic disease, however, is more prone to cause absorption of the walls, and consequently gives rise to the characteristic parchment or crackling sounds upon pressure.

Another class of diseases to which this cavity is subject, is the malignant tumors, especially the small round cell sarcoma, which originate so frequently in this cavity, and which is brought under the attention of the dental surgeon in its early stages more frequently than the general surgeon, who is apt to see it later, when remedial measures are well nigh useless, as instanced in a case during last August, in which I removed a superior maxillary bone for sarcoma, which had originated in the antrum, but had expanded the walls of this cavity, and forced itself into the surrounding tissues, but after removal it returned again in a very short time.

The need of early diagnosis in all diseases of the antrum is of great import to the sufferer, as many of these cases have gone on for years without relief. The diagnosis is more easily arrived at from exclusion than from any positive symptoms; and in all cases in which the diagnosis has been obtained, treatment should be instituted; and the first object is to obtain an entrance into the cavity; this is best effected through the anterior wall of the antrum, unless there be decayed teeth which can be extracted and an entrance then gained through the alveolus of the extracted tooth or teeth; however entrance may be gained, the cavity should be washed out in cases of Empyema with warm fluids containing mucilagenous or mild astringents. A good method of washing the cavities is by the Eustachian catheter, with a small rubber tube at the end, and this attached to the nasal douche, which will force the fluid into the nose. Of course in discovering any foreign body or growth, Papilloma or otherwise, the removal of this would be incumbent.

*Dr. Jay.*--I think the paper an excellent one, and very much to the purpose, also the remarks of Dr. Knight. I recollect extracting the fang of the first bicuspid from the jaw of a lady; she bled more freely from the nose than from the cavity where the tooth came out.

*Dr. Smith.*—A case reported for advice. It was something like this: in the extraction of the superior molar the root was broken, and in the attempt to remove it, it was forced into the antrum; the case was neglected for a few weeks, and the patient acquired the habit of taking water into the mouth and permitting it to pass out through the nose. Another annoyance was that water and fluids taken with meals would in part pass through the opening in the alveoli, into the antrum and out through the nose. I was consulted as to what could be done. I suggested that an attempt be made to recover the broken tooth. Have any gentlemen anything to say in regard to this particular case?

*Dr. Knight.*—If the opening is of sufficient size I would suggest the insertion of a small probe to see if the root could not be recovered, and then close up the opening into the antrum so that food and liquids could not enter from the mouth.

*Dr. Junkerman.*—I think in all cases where these accidents occur, and they do occur occasionally, if the roots are left in, they will at times become a source of trouble, and chances are they will do harm at some time. A bullet shot into the tissues may become encisted, but we get no encistment of tooth roots. If the antrum is larger than the root forced into it, and if the roots are ragged or rough, you are more likely to have trouble than if they are smooth. In regard to this case, if the cavity of the antrum be small there will probably be no trouble. If the cavity is large, the change of position, etc., will cause an irritation that will give considerable trouble, and the root will have to be removed. Of course, we should always get rid of this root if possible.

*Dr. Smith.*—During the washing of this cavity what assurance has the operator that the root has not floated out? There are certain instruments for the removal of roots, but none in my judgment suitable.

Dr. Fletcher also spoke of a case he had had quite recently, in which the odor was perceptible to the patient, but he was unable to detect it.

